# Exploring the role of vocalizations in regulating group dynamics

**DOI:** 10.1098/rstb.2023.0183

**Published:** 2024-06-23

**Authors:** Bing Xie, Josefine B. Brask, Torben Dabelsteen, Elodie F. Briefer

**Affiliations:** ^1^ Behavioural Ecology Group, Section for Ecology and Evolution, University of Copenhagen, Copenhagen, 2100, Denmark

**Keywords:** acoustic communication, group dynamics, collective movement, fission–fusion dynamics, vocal networks, social networks

## Abstract

Because of the diverging needs of individuals, group life can lead to disputes and competition, but it also has many advantages, such as reduced predation risk, information sharing and increased hunting success. Social animals have to maintain group cohesion and need to synchronize activities, such as foraging, resting, social interactions and movements, in order to thrive in groups. Acoustic signals are highly relevant for social dynamics, some because they are long-ranging and others because they are short-ranging, which may serve important within-group functions. However, although there has been an increase in studies concentrating on acoustic communication within groups in the past decade, many aspects of how vocalizations relate to group dynamics are still poorly understood. The aim of this review is to present an overview of our current knowledge on the role of vocalizations in regulating social group dynamics, identify knowledge gaps and recommend potential future research directions. We review the role that vocalizations play in (i) collective movement, (ii) separation risk and cohesion maintenance, (iii) fission–fusion dynamics, and (iv) social networks. We recommend that future studies aim to increase the diversity of studied species and strengthen the integration of state-of-the-art tools to study social dynamics and acoustic signals.

This article is part of the theme issue ‘The power of sound: unravelling how acoustic communication shapes group dynamics’.

## 1. Introduction

Acoustic communication plays an important role in the lives of social animals via its far-reaching effects on survival, reproduction and social relationships. Acoustic signals can convey information about the sender (e.g. species, identity, sex, age, size, condition and motivational state [1]), the behavioural context (e.g. foraging and movement [2]) and even the presence of specific objects, events or predators in the environment in some species [3,4]. As a result, the auditory modality is used in various aspects of animals’ social lives, such as mating, predation, territorial defence, parent–offspring interactions, social relationships and coordination of group movement [1].

Acoustic communication between one sender and one receiver, as a dyadic relationship, has been widely studied among social animals, while focusing for example on mate choice [5,6] or mother–infant interactions [7,8]. However, in social groups, communication occurs in an environment composed of many potential senders and receivers [9,10]. This can affect the behaviour of signallers and change the conditions of signalling. For example, ‘audience effects’, where signallers behave differently depending on the presence of listening conspecifics, have been shown to occur in several species [11–13]; and ‘eavesdropping’ [10] (sometimes referred to as social eavesdropping [14]), where a receiver gathers relative information about other interacting individuals by listening to their signal exchange, alters the conditions and constraints acting on signalling [9,10,15]. While these specific aspects of the relationship between vocal communication and sociality have been quite extensively investigated, many aspects are still not well understood. This includes the role that vocal communication plays in regulating the social dynamics within groups.

In the past two decades, there has been an increase in studies focusing on communication occurring within groups [1,9,16–18]. Furthermore, during the same period, the field of social behaviour has developed considerably, partly due to new techniques for the measurement and analysis of social behaviour [19–21]. We are therefore now at an exciting point in time, when the connection between vocal communication and sociality can be studied with state-of-the-art tools in both fields of research (bioacoustics and sociality), and with much yet to be discovered.

The aim of this review is (i) to provide an up-to-date and critical overview of the state of knowledge on the role that vocalizations play in social group dynamics, (ii) to highlight gaps in the understanding of this topic, and (iii) to suggest directions in which this field of research could develop. Our review focuses on four themes that cover different aspects of group dynamics: collective movement, separation risk (i.e. cohesion maintenance), fission–fusion dynamics and social networks. These four topics are biologically strongly interconnected and thus do not correspond to fully separate biological aspects but rather to different research themes. The review is based on a literature search concerning these themes and their relation to acoustic communication. We will discuss a broad range of taxa, while mainly focusing on mammals and birds living in social groups, as this is what most papers we have reviewed studied, and because we decided to focus only on airborne sounds in this review [22,23]. Many insect species are typically social animals but rely on substrate vibrations to communicate (e.g. termites, ants and social bees [24]), and these are therefore not extensively considered here.

In the following, we first provide a brief overview of our literature search (§2). We thereafter consider each of the four themes and provide a review of relevant studies from the literature about their link to acoustic communication (§§2.1–2.4). This is followed by a consideration of research gaps and future directions (§3) and concluding comments (§4).

## 2. Current knowledge about the role of vocalizations in shaping social group dynamics

**Figure 1.**
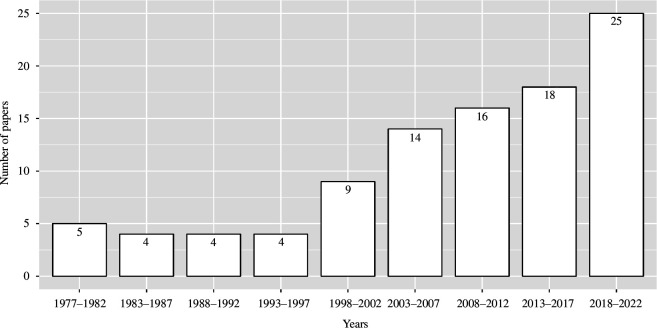
Papers published per 5-year period on the role of vocalizations in group dynamics.

We based our review on a collection of articles that we have gathered via Google Scholar using the following search terms, where the search was applied to all parts of the text (i.e. the standard of this search engine): (collective movement OR group cohesion OR decision making OR vocal network OR social network OR separation risk OR fission–fusion) AND (vocal OR acoustic). We extracted articles related to the topic and tracked additional references using a snowballing approach to complete the database by searching studies that referenced or were referenced by the extracted studies. In total, we gathered 99 articles. These articles are listed in electronic supplementary material, table S1 [25], along with a mention of whether they are based on observations or experiments (e.g. playbacks and laboratory settings). The number of papers published on the role of vocalizations in group dynamics increased from five to nine per 5 years between 1977 and 2002, followed by over 14 articles per 5 years after 2003 (figure 1). Particularly, this number grew rapidly after 2003. There has since been an increased interest in this topic during the past 20 years, but still with a limited number of papers published per year. We note that our paper search is unlikely to return all papers relevant to the topic, and our conclusions are based on the papers included in the review. Our collection of articles is not meant to be an exhaustive list of papers, but to show development trends in this field of research.

### (a) Vocalizations and collective movement

A *collective movement* has been described as a group of individuals departing synchronously, moving in the same direction and maintaining cohesion during movement, until they stop [26]. It specifically implies spatial displacement resulting from mediated individual decisions, which relies on behavioural signals or external cues (i.e. the presence and behaviour of predators) to drive responses [26]. A collective movement consists of several steps, including a pre-departure period (a period to reach some kind of consensus on timing and direction for movements [27]), the initiation of movement, joining and movement of all individuals, staying together during movement and termination of movement [26,28,29]. Group-living animals heavily rely on signals to transfer information among group members [26], and vocalizations, with their potential multi-receiver benefits, have been shown to be exchanged frequently during collective movement. Across the articles that we have gathered, the connection between vocal signals and collective movement is one of the most popular research topics. In total, 33 out of the 99 articles that we collected focused on the role of vocalizations in group movements, including their role during the initiation of movements (25 papers), as well as their influence on other aspects of group movements, such as the direction, speed and distance travelled (8 papers; electronic supplementary material, table S1).

Based on their acoustic parameters, vocalizations can be classified into different types, which usually serve different functions (e.g. contact calls vs alarm calls). During movement, the types of vocalizations emitted will depend on the stage of the movement. For instance, chimpanzees (*Pan troglodytes*) were observed to use ‘travel hoos’ to facilitate the initiation of group movement [30], while they use ‘pant-hoots’ to maintain contact with conspecifics and ‘rest hoos’ to extend the resting time and decrease the likelihood of moving [31]. Likewise, Central American squirrel monkeys (*Saimiri oerstedii*) trigger troop movements in the wild by producing an increased proportion of ‘twitters’, but a decreased proportion of ‘bent mast chucks’ [2]. Finally, meerkats (*Suricata suricatta*) use ‘lead calls’ to initiate group movements and ‘move calls’ to change foraging patches in the field [32].

During movement initiation, several parameters of vocalizations, such as their rate of utterance, intensity or frequency, can play a role. In African wild dogs (*Lycaon pictus*), a significant difference in the total number of ‘sneezes’ produced during successful versus unsuccessful initiation of group movement was found [33]. Moreover, red-fronted lemurs (*Eulemur rufifrons*) were observed to produce higher rates of ‘grunts’ during the pre-departure period than during foraging [34]. Among birds, mass departures of jackdaws (*Corvus monedula*) were found to be governed by an increase in calling intensity (calculated through power spectral density) and they also departed earlier as a reaction to playbacks of ‘roost calls’ compared to wind noises [35]. Similarly, domestic geese (*Anser domesticus*) were also recorded to utter a higher number of calls to gather more followers during initiation [36]. Finally, in insects, honey bee (*Apis mellifera*) swarms were shown to emit a high-pitched ‘piping’ vibration near take-off [37,38], and specifically to raise the fundamental frequency of ‘piping’ as a preparation to lift-off to fly to a new home by ‘quorum sensing’ (i.e. when a certain threshold of minimal number of individuals conducting a behaviour is reached, the probability of other group members performing the same will rise sharply) [39,40].

Vocalizations have also been shown to shape group movement directions. Colombian white-faced capuchins (*Cebus capucinus*) were recorded to produce ‘trills’ at higher rates when being positioned at the forefront of troops compared with other positions in the troop, and to alter directions of troop movements by emitting ‘trills’ at the side or back of the group [41]. Likewise, meerkats adjust group movement directions while maintaining cohesion by following vocal hotspots (i.e. many meerkats uttering high rates of calls), as shown during a playback experiment [42]. Moreover, it seems that flight calls can be used in birds for orientation, especially when visibility is poor, to reduce dispersion and avoid collisions (see [43] for a review). For example, migrating birds (of unknown species) were shown to change flight paths following ‘flight call’ playbacks [44]. Zebra finches (*Taeniopygia guttata*) were also recently found to use vocalizations to adjust positions and avoid collisions in flight [45].

Finally, vocalizations can affect other aspects of group movement, such as travel distance and speed. During playbacks of greater spot-nosed monkey (*Cercopithecus nictitans*) calls, the length of a call sequence was found to affect how listeners react, with a longer sequence resulting in a longer group movement distance [46]. Group speed and probability of changing foraging patches dramatically increased when the number of meerkats emitting ‘moving calls’ increased from two to three [47]. Likewise, ultrasonic vocalizations emitted by female mice (*Mus musculus*) were shown to significantly accelerate a male’s travelling speed, compared to the presence of a silent female [48].

Overall, our review of the literature based on the search terms mentioned above shows that vocal signals play an important role in regulating group movement in a range of species. Specific vocalization types, vocalization rate or other vocalization features (e.g. frequency) have been shown to be associated with pre-departure periods, departure periods and collective movement periods, as well as with changes in movement direction, speed and the distance travelled. Our collection did not contain any articles that focused on how vocalizations influence termination of group movements, although some articles suggested that vocal signals could affect this step [28,49,50]. In that situation, a cessation of vocal signals might also trigger movement termination.

### (b) Vocalizations and separation risk

Staying together as a group can be challenging. Activities such as foraging entail *separation risk* and make cohesion maintenance more challenging, resulting in an effort for groups to synchronize and coordinate in order to mitigate the risk and maintain cohesion [51,52]. Over the past 20 years, researchers have been interested in the mechanisms that help mitigate separation risk and maintain group cohesion, with a primary focus on human (*Homo sapiens*) societies [53,54], and increasing interest in numerous animal species [55–57]. When social animals move for foraging or travelling, they risk being spatially separated and losing contact with one another [58]. To maintain cohesion, they can produce vocalizations to keep in contact, locate lost members and subsequently reunite [59]. Out of the 99 collected papers, 20 focused on the role of vocal signals in mitigating separation risk and maintaining cohesion, where separation risk was typically assessed based on the distance between individuals or visibility (e.g. forest habitat structure [60]; electronic supplementary material, table S1). Cases that focus on different vocal behaviours involving distinct distance or visibility conditions, with or without a consequence (i.e. reunite), will be discussed in this section (for cases concentrating on group composition changes (i.e. reunite, recruit, split and member exchanges), please refer to the next section (§2c. Vocalizations and fission–fusion dynamics).

‘Contact calls’ are the main type of affiliative vocalizations used across species to maintain contact with conspecifics, thus functioning to mitigate separation risk and playing a role in fission–fusion dynamics (§2c) [57]. African bush elephants (*Loxodonta africana*) exchange ‘contact calls’ between family members to locate each other and reunite [61], and the production of these ‘rumbles’ results in a net decrease in distance between individuals in the wild [58]. In goats (*Capra aegagrus hircus*), peripheral individuals belonging to a semi-free-ranging group showed higher probabilities of producing contact calls than animals in the centre, and the production of these calls was found to be associated with the end of the group expansion, followed by group contraction [62].

A vocal feature that often changes in situations of separation is call rate. Free-ranging baboons (*Papio cynocephalus*) often utter loud ‘contact barks’, spontaneously or in response to playbacks of these same barks, at higher rates when they are at risk of losing contact with the main body of the group [63]. Likewise, ring-tailed lemurs (*Lemur catta*) were observed to give higher rates of ‘meow calls’ when group members were absent from the surroundings compared to when they were present [64], and during behaviours that might lead to separation [65]. Similarly, Diana monkeys (*Cercopithecus diana*) were found to emit higher rates of ‘clear calls’ when the group was spread over a larger area, and these calls were answered by similar ‘clear calls’, especially by out-of-sight group members [66]. Finally, in a natural habitat with poor visibility, Japanese macaque (*Macaca fuscata*) call rates were observed to be higher than in a good visibility habitat [67].

The duration and frequencies of vocalizations can also be related to the risk of separation and cohesion maintenance. Japanese macaques were observed to produce ‘coo calls’ with different acoustic features depending on their proximity to group members; the fundamental frequency, its modulations and the call duration increased with separation distance, making these sounds more locatable [68]. In a similar way, when ring-tailed lemurs are distant from each other in the wild, they produce vocalizations with shorter durations, greater frequency ranges and higher median frequency, which likely increases the chance of locating the sender [64].

When acoustic signals propagate over long distances, they are subject to attenuation and distortion [69]. To satisfy both short-range and long-range communications, social animals have evolved different types of calls for communication over short versus long distances with various acoustic features to modify the potential of sounds to propagate (i.e. lower frequencies, longer durations and higher sound pressure level (SPL) for improving long-distance communication), and to adjust detectability and localizability (i.e. repetitive, sharp onsets, wide-frequency range, frequency modulations and amplitude modulation for improving localizability were suggested for long-distance communication) [70–74]. Accordingly, spinner dolphins (*Stenella longirostris*) were observed to produce different call types at different distances: ‘whistles’ play a specific role in maintaining long-distance contact, while ‘burst pulses’ are produced by individuals at closer distances [55]. Similarly, wild pygmy marmosets (*Cebuella pygmaea*) emit different ‘trills’ depending on the distance, with the most localizable ‘trills’ being produced when animals are far apart and the least localizable ‘trills’ being produced when individuals are closer together [70].

To conclude, the studies reviewed here show that vocalizations facilitate group cohesion and shape group dynamics by preventing dispersed individuals from being separated from the rest of the group, and by allowing them to contact, relocate and rejoin the group. In addition, animals that are separated from their group usually utter vocalizations at a higher rate, with higher fundamental frequencies, more frequency modulations, longer durations and higher SPL, some of which make them more localizable from a distance. These changes could also partially reflect a higher level of arousal in the producer due to stress related to social separation [75]. Animals also often use different call types as a function of the separation distance, to deal with the consequences of sound propagation.

### (c) Vocalizations and fission–fusion dynamics

The term ‘fission–fusion dynamics’ describes processes where subgroups split and merge over time, leading to temporal changes in group composition and size [76]. This provides an ideal setting to study how individuals regulate group dynamics [58,77]. Many species use vocalizations to facilitate fission–fusion dynamics, and more specifically, during splitting and reunion of sub-groups. Among all 99 articles that we found on the role of vocalizations in group dynamics, 37 of them focused broadly on the role of vocalizations in fission–fusion dynamics (including intra-group structure dynamics, member exchanges and intra-group movements, attraction and recruitment of individuals to groups; electronic supplementary material, table S1).

The role of vocal signals in regulating fission–fusion dynamics has mostly been investigated in primates. Male chimpanzees were observed to emit more ‘pant hoots’ before compared to after fusion with other males, suggesting that these calls facilitate the fusion stage of the subgroup [78]. They were also found to emit special ‘pant hoots’ with varying fundamental frequency, tonal quality and the presence of a ‘let-down’ phase (the last phase of ‘pant hoots’, consisting of a short series of inhalation/exhalation with decreased amplitude and frequency over time), before a small subgroup joined others [79]. Similarly, bonobos (*Pan paniscus*) were observed to produce more calls directed at both males and females during the fusion stage of the group rather than the fission [80], and the production of a ‘low hoot + high hoot’ combination was more likely to result in inter-subgroup fusion than ‘high hoot’ alone [81]. Moreover, white-bellied spider monkeys (*Ateles belzebuth*) tend to respond to a ‘loud call’ from distant group members and approach that call rather than avoiding or ignoring it, which results in new members joining the subgroup and the subgroup size increasing within an hour after calling [82].

Other mammals also use vocalizations to attract conspecifics and facilitate reunion. Elephants were found to utter low-frequency ‘rumbles’ during reunions and in close proximity, implying multiple functions for this call in social communication [83]. Likewise, dwarf mongooses (*Helogale parvula*) are more likely to respond by looking at and approaching a loudspeaker in response to playbacks of ‘movement calls’ than ‘close calls’, suggesting a recruitment function of ‘movement calls’ [84]. Moreover, pied babblers (*Turdoides bicolor*) were found to use calls with different structures to coordinate two different forms of recruitments—‘come to me’ and ‘come with me’—and playbacks confirmed the observation by attracting more individuals to the speaker via ‘come to me’ calls compared to ‘come with me’ [85]. Many bats live in societies with fission–fusion dynamics and use ‘contact calls’ to maintain contact with conspecifics and recruit group members [86]. Spix’s disk-winged bats (*Thyroptera tricolor*) were found to produce two social calls, ‘inquiry calls’ and ‘response calls’, to recruit groupmates to leaf roosts. Most of the flying bats were observed to enter the roosts [19] and to spend significantly less time finding the roost when the bats in the roost produced ‘response calls’ at higher rates [87]. Likewise, noctule bats (*Nyctalus noctula*) reacted more strongly to playbacks of ‘social calls’ emitted from roosts compared to no calls or background noises, by landings and inspections when returning from foraging trips, highlighting the importance of ‘contact calls’ for locating conspecifics in roosts and facilitating reunion [88].

One well-studied type of vocalization that plays a role in group fusion is ‘foraging calls’, which serve to attract and recruit group members to feeding spots [89]. Additionally, these types of calls may serve in a wider range of social contexts [90]. Many species have been observed to utter ‘foraging calls’, resulting in group recruitment, such as Geoffroy’s spider monkeys (*Ateles geoffroyi*) [91], willow tits (*Poecile montanus*) [92] and greater spear-nosed bats (*Phyllostomus hastatus*) [93]. In cliff swallows (*Hirundo pyrrhonota*), for instance, ‘foraging calls’ are used to attract conspecifics, but also during fission–fusion events. When loose groups away from colonies produce these calls, this elicits a dense group for several minutes, which then dissolves or breaks up [90]. Moreover, orange-fronted conures (*Eupsittula canicularis*) in foraging flocks were shown to follow and fusion more often with leaders of interactions involving contact calls, and to respond with a higher rate of calls to playbacks of calls from vocal leaders than vocal followers [94]. Pied babblers were found to emit ‘purr calls’ in both food and no-food contexts. These birds were observed to recruit more members by giving a ‘purr call’, than by giving a ‘contact call’ or remaining silent, and they also responded to playbacks of ‘purr calls’ by approaching the speaker [95]. Nestlings of this species responded to playbacks of adult ‘purr calls’ via begging while fledglings approached the speaker [96]. Additionally, this species uses another type of call, ‘chucks’, to keep distance from one another, and individuals stay farther away during playbacks of ‘chucks’ [97].

In summary, the reviewed studies show that vocalizations can have important functions in group fission–fusion events. Animals often uze different call types, call combinations and different call features (e.g. fundamental frequency) to answer signallers, resulting in the regulation of member exchanges and intra-group movements, such as splitting and reunion.

### (d) Vocalizations and social networks

The ‘social network’ of a population is the pattern of social connections between individuals, i.e. the fine-scale social structure. Here, individuals constitute the nodes of the networks and their social relationships constitute the edges. Social networks can be analysed quantitatively by social network analysis [21,98,99]. We note that the term ‘communication networks’ (i.e. individuals within signalling range) has been used with a different meaning [9] (for a distinction between social and communication networks, please refer to [100]). In the field of animal behaviour, social network analysis has mostly been used to study social structures based on physical interactions or spatial proximity between individuals [96]. It is, however, also possible to construct networks where the links are based on vocal communication (e.g. vocal interactions or vocal similarity) between individuals. We here generally refer to such networks based on vocal communication as ‘vocal networks’ (see [16,100] for general consideration of the connection between vocal communication and social networks). Vocal networks can be considered as a type of social structure, and can be compared with social networks based on physical interactions or spatial proximity in order to investigate the link between vocal communication and social relationships. We found 14 papers that investigated the interaction between vocal networks and social dynamics. Out of these 14 papers, 12 of them were published in the past decade (electronic supplementary material, table S1), highlighting the novelty of this area. These studies involved building networks based on vocal exchanges (hereafter ‘vocal exchange networks’), proximity between senders and receivers (hereafter ‘proximity-based vocal networks’) or similarities in the acoustic structure of the vocalizations of individuals (hereafter ‘vocal similarity network’). These resulting ‘vocal networks’ were further compared with social interaction networks, proximity networks or geographical structures.

Vocal exchanges can be considered as a form of social interaction. For instance, affiliative vocalizations have been hypothesized to be used to maintain social relationships within groups, in the same way as other types of affiliative interactions (e.g. grooming), but with the advantage that these interactions are less time consuming, not restricted to close proximity and effective at a larger distance than physical interactions [101]. These exchanges can thus function to form and maintain social bonds. Many species have been reported to interact vocally more with partners that are socially closer. For instance, Guianan squirrel monkeys (*Saimiri sciureus*) were observed to exchange ‘chuck calls’ with shorter latency and more often with group members with whom they had more affiliative behaviours [102]. They are also more likely to respond to playbacks of these affiliated individuals or to unfamiliar calls with similar acoustic structure to their own group members, compared to less affiliated individuals or less similar calls [103]. Similarly, zebra finches were shown to preferentially build vocal bonds with their mates, and secondarily with their nearest neighbours [104].

Vocal exchange network studies have revealed that vocalizations can also function to strengthen weak social associations or reshape social interactions. In male bottlenose dolphins (*Tursiops truncatus*), higher rates of call exchanges were observed between individuals sharing weaker social bonds (assessed based on proximity), while strong social bonds were instead maintained through higher rates of affiliative interactions [105]. Similarly, chimpanzees were found to build weak social bonds (assessed through a mix of eight behaviours) through vocal exchanges, but strong social bonds through gestures and bimodal signals (vocalization and gestures) [106]. Their vocal exchange network was also observed to be positively related to their proximity network, and was suggested to allow them to communicate with more individuals than through gestures, proximities or grooming alone [107]. Additionally, in ring-tailed lemurs, individuals were observed to interact with more members in the call exchange network than in the grooming network [64], and individuals only responded vocally to individuals they frequently groomed, rather than to all grooming partners [108].

Vocal exchange networks have been found to be a useful tool in studying group stability. The structure of vocal exchange networks was found to remain stable in Emei music frogs (*Babina daunchina*), when comparing the structures before and after playbacks [109]. Likewise, zebra finch vocal exchange networks were shown to be stable across days [110]. In addition, siamang (*Symphalangus syndactylus*) vocal exchange networks, which were built based on vocal interactions among different groups, showed that unstable groups were significantly more likely to receive call responses from neighbouring groups, compared to groups with stable structures [111].

Proximity-based vocal networks are another example of vocal networks that can be used to investigate the function of vocalizations in regulating social dynamics. In white rhinoceros (*Ceratotherium simum*) four different proximity-based vocal networks were measured (with individuals situated less than one body length away being considered as potential receivers of the sounds), respectively, based on four call types. This study not only revealed negative relationships between agonistic vocal networks (‘grunt’ and ‘hiss’) and social networks (measured as time spent in close proximity) of females, but also suggested that different call types are linked to different interaction networks (i.e. the ‘pant’ network was positively correlated with the defensive interaction network, while the ‘hiss’ network was positively correlated with the aggressive interaction network) [112].

The acoustic features of vocalizations can, in the same way as the rate of vocal exchanges, be related to social bonds. For example, the similarity between individuals in call features was found to be a strong indicator of social bonds in Campbell’s monkeys’ (*Cercopithecus campbelli*), as displayed by a significant relationship between grooming interactions and acoustic similarities [113]. Networks built on acoustic similarities (i.e. ‘vocal similarity networks’), which have so far only been investigated in a few studies, also have the potential to reveal the spatial structures of populations. When vocal similarity network analysis was applied to 11 populations of Doria’s Asian treefrog (*Chiromantis doriae*), three clusters were identified in the network that were partially associated with geographical distances among different populations [114]. In another study, a song-sharing network in silvereyes (*Zosterops lateralis*) indicated that populations at similar longitudes tended to share more syllables than at similar latitudes, and certain sets of syllables were more likely to co-occur than others [115].

To summarize, the reviewed studies show that vocalizations can strengthen social bonds and complement other types of social interactions. Various types of vocal networks (vocal exchange networks, proximity-based vocal networks and vocal similarity networks) have been quantified and can serve as useful tools to study the relationship between vocal communication and social dynamics.

## 3. Research gaps and future directions

In the following, we suggest potential directions and focus points for future research on the connection between vocalizations and social dynamics, based on the reviewed literature.

Overall, the orders that occurred most frequently in the reviewed studies were the Primates (41 articles), followed by the Passeriformes (18 articles), in total constituting 60% of the articles we collected. Studies on other orders all amount to fewer than 10 each, with a relatively small number of articles for Carnivora (seven articles), Chiroptera (six articles), Hymenoptera (six articles), Artiodactyla (five articles) and very few articles (fewer than five articles) for other mammals, other birds and amphibians.

Primates have likely been the focus of many studies on vocalizations and group dynamics because they are highly social, show great variation in social structure and are intensively vocal [116]. This makes them excellent subjects, as our closest relatives, to investigate the evolution of vocal communication and the precursors of human language [117,118]. Similarly, Passeriformes are highly vocal and social (especially the Corvidae and Paridae families) and have extensive vocal learning abilities, making them perfect subjects to explore the complexity of links between vocalizations and social dynamics [119].

Surprisingly, we only found a few studies on other species that share similar features (both vocally and socially). For example, our sample only contained one article focusing on the largest mammalian order, rodents, which are characterized by widespread sociality (70 social species for 39 genera [120]) and extensive vocality [121]. Likewise, the second largest order of mammals—bats—for which our sample contained only six papers, comprises about 20% of all mammal species and they are highly vocal with rich vocal complexity and diverse social organization [86,122]. Additionally, many species of Artiodactyla (e.g. whales [123], deer [124]), Carnivora (e.g. wolves [125], the Hyaenidae [126], lions [127]), Equidae [128], Anseriformes and Charadriiformes [129], Psittaciformes [130] and Amphibia [109] share similar interesting features, but the studies we found are sporadic. Although the lack of these species from the literature could potentially be partly due to the methodology of the search, it suggests that more attention on these species could be beneficial.

In terms of vocal features that the studies considered, most of them were primarily focused on the occurrence of vocalizations, the types of vocalizations and vocalization rate, in total accounting for about 72% (71/99) of all articles. These features are relatively easy to assess without detailed knowledge of acoustics or dedicated software, which may explain this bias. Studies considering other call features, such as frequencies, duration of calls, call amplitude or loudness, call sequence, call combinations and call similarities were scarce in our sample. This suggests that further studies on the relationship between these vocal features and sociality are needed.

The effect of vocalizations on group movement has been well studied, occupying 33% (33/99) of the total number of papers that we reviewed, among which the impact of vocalizations on group movement initiation represented 76% (25/33). Our literature search found few articles on how vocal signals influence group movement distance, direction and speed (e.g. [41,42,46,47]), and no papers on how vocalizations, or decreases in vocal production, affect termination of group movements. We thus suggest that further studies should focus on these rarely studied but likely important aspects.

Studies on the role of vocalizations and separation risk or cohesion maintenance amount to 20% (20/99) of all articles collected. Despite the likely importance of vocalizations in minimizing separation risk and maintaining proximity for group living species, our paper search only found three articles published in the past decade, and an average of seven articles published in the previous two decades. However, the link between vocalizations and fission–fusion dynamics, in total amounting to 37% (37/99) of all articles collected, were more popular through all years, even before the twenty-first century (10 articles). Foraging calls contributed to half of the fission–fusion papers, making them a popular theme of social recruitment. Many other specific call types, such as mobbing calls [131,132], alarm calls [133,134] and cooperative hunting calls [135], remain to be more clearly integrated within fission–fusion dynamics studies.

We found 14 studies on vocal networks, and this topic seems to have gradually attracted more attention in the past decade (with 12 of the studies being from the past decade). Analysis of vocal networks based on various aspects of interactions or acoustic structure (e.g. vocal exchanges and vocal similarity) has been used to investigate the relationship between vocal signalling and other types of social interactions, association strength and the temporal dynamics and spatial structures of populations, and there are many more possible applications [16,17]. Given the importance of vocalizations for social relationships, it seems that more studies on vocal networks, and their link to other types of social networks, could be highly relevant for our understanding of social systems.

Finally, slightly more than half of the studies we found (56%, 55/99) were observational (electronic supplementary material, table S1). When possible, we recommend conducting experiments following observations to enable the investigation of causal directions in the complex relationship between vocal signals and group dynamics. For example, playback experiments, in which specific sounds are played back from a loudspeaker, can be used to investigate whether a given call-type functions to repel or attract individuals [97]. These methods can therefore be useful for examining causal links.

In summary, our review implies that interest in the link between vocalizations and group dynamics is quickly growing. It also indicates that studies we reviewed on this topic mainly focused on a restricted number of orders and species and on specific features of vocal signals. We would like to encourage more research on species belonging to other orders than the Primates and Passeriformes, as this could be helpful for deciphering how specific parameters of sounds influence group dynamics and social relationships across species and taxa. We also recommend focusing more on sound acoustic structure, termination of collective movement, separation risk (cohesion maintenance) and vocal networks.

## 4. Conclusion

Our knowledge about the link between vocal communication and group dynamics that we have summarized above clearly indicates that vocalizations play an important role in shaping social group dynamics in various manners. Animals use specific call types, call rates and sequences of different call types in a way that implies that vocalizations enhance the success rate of initiation of group movements. Different vocal features are also used to coordinate and adjust group movement characteristics, such as travel distance, direction and speed. In addition, together with the above-mentioned vocal features, animals utter calls with different durations and in different combinations to mitigate separation risk, facilitate cohesion and regulate fission–fusion dynamics. Moreover, the studies we mentioned imply that using social network analysis to study vocal networks is a useful approach to investigate vocal communication from a social perspective that warrants more attention. Our review also implies that, while a number of important insights into the link between vocal communication and group dynamics have already been obtained, there is much left to discover before we reach a comprehensive understanding of sociality. We hope that this review and the suggestions for future research directions will be useful in the further development of this exciting research area.

## Data Availability

Supplementary material is available online [136].
